# Design of Functionally Stacked Channels of Oxide Thin-Film Transistors to Mimic Precise Ultralow-Light-Irradiated Synaptic Weight Modulation

**DOI:** 10.3390/mi13040526

**Published:** 2022-03-26

**Authors:** Ji Sook Yang, Sung Hyeon Jung, Dong Su Kim, Ji Hoon Choi, Hee Won Suh, Hak Hyeon Lee, Kun Woong Lee, Hyung Koun Cho

**Affiliations:** 1 School of Advanced Materials Science and Engineering, Sungkyunkwan University (SKKU), 2066 Seobu-ro, Jangan-gu, Suwon 16419, Gyeonggi-do, Korea; yangtwin1@naver.com (J.S.Y.); wjdtjdgus2@skku.edu (S.H.J.); dskim2846@naver.com (D.S.K.); jeehun_choi@naver.com (J.H.C.); naekkeo@skku.edu (H.W.S.); zadxs@skku.edu (H.H.L.); leekunwoong1@naver.com (K.W.L.); 2 Research Center for Advanced Materials Technology, Sungkyunkwan University (SKKU), 2066 Seobu-ro, Jangan-gu, Suwon 16419, Gyeonggi-do, Korea

**Keywords:** artificial photonic synapse, thin film transistor, amorphous oxide semiconductor, ultralow light intensity, stacking structure

## Abstract

To utilize continuous ultralow intensity signals from oxide synaptic transistors as artificial synapses that mimic human visual perception, we propose strategic oxide channels that optimally utilize their advantageous functions by stacking two oxide semiconductors with different conductivities. The bottom amorphous indium–gallium–zinc oxide (*a*-IGZO) layer with a relatively low conductivity was designed for an extremely low initial postsynaptic current (PSC_i_) by achieving full depletion at a low negative gate voltage, and the stacked top amorphous indium–zinc oxide (*a*-IZO) layer improved the amplitude of the synaptic current and memory retention owing to the enhancement in the persistent photoconductivity characteristics. We demonstrated an excellent photonic synapse thin-film transistor (TFT) with a precise synaptic weight change even in the range of ultralow light intensity by adapting this stacking IGZO/IZO channel. The proposed device exhibited distinct ∆PSC values of 3.1 and 18.1 nA under ultralow ultraviolet light (350 nm, 50 ms) of 1.6 and 8.0 μW/cm^2^. In addition, while the lowest light input exhibited short-term plasticity characteristics similar to the “volatile-like” behavior of the human brain with a current recovery close to the initial value, the increase in light intensity caused long-term plasticity characteristics, thus achieving synaptic memory transition in the IGZO/IZO TFTs.

## 1. Introduction

Since the human brain is capable of high integration and high-efficiency information processing, neuromorphic computing has attracted interest as a next-generation computing system that can replace the existing von Neumann computing [1,2]. In the human nervous system, the brain contains ≈10^11^ neurons and ≈10^15^ synapses, which are essential for processing complex problems, and the neuromorphic system combines memory and computation [3]. In neuroscience, synaptic plasticity fundamentally supports the brain in processing incoming information at high efficiencies and performing parallel operations [4,5]. Therefore, it is crucial to mimic synaptic functions to implement neuromorphic computation systems with proper devices. Artificial synaptic devices, in which the synaptic weight can be controlled using external stimuli, have been reported to perform basic synaptic functions [6,7].

Over the past few decades, two-terminal devices have been explored as the building blocks of neuromorphic systems, such as resistance random access memory (RRAM) [8,9], ferroelectric memory [10,11], and phase-change memory (PCM) [12,13]. Although these two-terminal devices have demonstrated excellent capacity for basic neural functions, the human brain contains more synapses than neurons, which implies that multiterminal devices are in high demand for complex functions. However, to conduct filament formation, two-terminal devices such as an RRAM encounter problems of sneak path current generation and high voltage requirement for write/read operations, causing high energy loss inconsistent with the high energy-efficiency aspect of neuromorphic computing [8]. Alternatively, in recent years, three-terminal thin-film transistor (TFT) synaptic devices, which are more similar to biological neural systems, have been proposed to realize synaptic functions [14,15,16,17,18,19,20]. The conductance of the proposed TFT synaptic devices can be continuously modulated by the third terminal using various input pulse conditions. Moreover, these devices can implement signal transmission and self-learning simultaneously and provide a platform for creating multiterminal circuits that can imitate biological neurons with many synaptic connections.

In recent years, indium–gallium–zinc oxide (IGZO)-based TFT devices have been developed to mimic synaptic functions [14,17,18,19,20]. *a*-IGZO has been commercialized in flat panel displays owing to its unique electron-transport properties [21,22,23,24]. Most synaptic devices using *a*-IGZO TFTs use electrical signals to stimulate the devices, which is similar to the action potential in biological systems [19,20]. In particular, the persistent photoconductivity (PPC) of *a*-IGZO can be used to mimic human memory characteristics; thus, it is widely applied in photonic synapse transistors for artificial visual systems [14]. Moreover, the human brain precisely controls synaptic weight, which is defined as the connection strength between the axon terminal and dendrites; thus, learning and memory behaviors appear differently under various light stimuli. The excitatory postsynaptic current (EPSC) is expressed as the change in synaptic weight by neurotransmitters released from the synaptic cleft. Thus, different intensities of light cause changes in synaptic weight, which affects the increase or decrease in the EPSC amplitude [25]. In addition, the gradual decay of EPSCs after light stimulation is removed. The ratio of the PSC_i_ to the remaining current owing to the PPC phenomenon is defined as the non-volatile memory of the light stimulus [26]. Biological synapses that can precisely control synaptic weight have different EPSC amplitudes and memory characteristics for a wide range of light stimuli. This implies that synaptic transistors mimicking biological synapses should achieve optimal synaptic plasticity over a wide range of light intensities.

However, there are still technical limitations to using *a*-IGZO TFTs as photonic synapse devices for artificial visual systems. As mentioned above, the *a*-IGZO film provides an effective pathway for the transport of photogenerated carriers as a channel layer with relatively prominent electrical conductivity. Thus, the optical synaptic characteristics of *a*-IGZO TFTs, such as short-term plasticity (STP) and long-term plasticity (LTP), have been demonstrated by controlling the input optical signals [14,17,18]. The reported photonic synapse devices have primarily focused on the modulation of synaptic plasticity using oxide-based transistors that can mimic the basic functions of biological synapses. Since most photonic synapse devices use only a high light intensity as the presynaptic stimulus, a single-layer *a*-IGZO TFT can easily obtain a distinguishable neuromorphic performance for the input light [14,17,18]. However, from the perspective of input light with an ultralow intensity [27], the *a*-IGZO channel layer does not have sufficient conductivity to discretize various input lights. Therefore, an increase in the conductivity of the channel layer is necessary to attain the ability to discriminate individual light intensities in a wide range of light stimuli, including ultralow intensities. However, the problem of suppressing the off-current is encountered when high-conductivity materials are used as the channel layer of TFTs [28].

In this study, we created a functional stacking structure of IGZO/IZO as synaptic transistors, which exhibited a high optical synaptic performance to mimic an artificial visual system. In the bilayer IGZO/IZO TFT system, both *a*-IGZO and *a*-IZO films were used as combined channels to achieve appropriate charge transport behavior and the designed memory characteristics, demonstrating the characteristics of an innovative synaptic device capable of effectively mimicking important synaptic functions such as EPSC, paired-pulse facilitation (PPF), STP, and LTP. The fabricated devices induce a distinct synaptic weight change even under low-intensity light irradiation from 1.6 to 8.0 μW/cm^2^, resulting in high-resolution EPSC for a wide spectrum of light. The consecutive synaptic weight changes of the artificial synapse in response to various stimuli can be considered as data treatment that is very similar to a biological synapse. This paper presents the possibility of simulating highly efficient human visual memory in neuromorphic computing systems by utilizing a photonic synapse transistor that can recognize successive synaptic weight changes under ultralow-intensity light.

## 2. Experimental Details

### 2.1. Fabrication of n-Type a-IGZO and a-IZO Thin-Film Transistors

*a*-IGZO TFTs with a bottom-gate configuration were fabricated on SiO_2_/Si substrates. Here, *a*-IGZO (In:Ga:Zn = 2:1:2 atomic ratio, 15 nm) and *a*-IZO (In:Zn = 7:3 atomic ratio, 15 nm) channel layers were deposited using radio-frequency sputtering (150 W, 3 mTorr, room temperature). For the *a*-IZO films, different Ar/O_2_ ratios of 30/0, 28/2, and 25/5 were used to control the electrical properties. The active channel layers were defined using conventional photolithography and a wet-chemical etching process (HCl/DI water = 1/5), which was followed by thermal annealing (200 °C, 1 h, air) in a furnace. Source/drain Mo (100 nm) electrodes were deposited by direct-current magnetron sputtering (65 W, 3 mTorr) at room temperature and defined using a lift-off process. The channel width (W) and length (L) of the fabricated *a*-IGZO and *a*-IZO TFTs were 500 and 50 μm, respectively.

### 2.2. Electrical Characterization

The electrical behavior of the transistors was characterized using 4145B (HP) and 4200-SCS (Keithley) semiconductor parameter analyzers at room temperature. To evaluate the electrical performance of the transistors from the transfer curve, the field-effect mobility (μ_FE_) and subthreshold swing (SS) were calculated using the equations $\mu_{FE}=\left[\frac{L}{WC_{ox}V_{D}}\frac{dI_{D}}{dV_{D}}\right]$ and $SS={\left[{\left(\frac{dlogI_{D}}{dV_{G}}\right)}_{max}\right]}^{-1}$, respectively. In addition, the threshold voltage (V_th_) was manifested in the (I_D_)^1/2^ − V_G_ plots. The V_th_ was determined by the constant current method (gate voltage at current level of L/W × 10 nA). Bias (V_G_ = −10 V) and illumination (UV light, 350 nm, 8.0 μW/cm^2^) stress tests were performed at constant V_D_ of 0.1 V. Bias (V_G_ = −10 V) and illumination (UV light, 350 nm, 8.0 μW/cm^2^) stress tests were performed at a constant V_D_ of 0.1 V. The synaptic behaviors were characterized using 4155C (HP) at room temperature with external UV light pulse (350 nm, 1.6–8.0 μW/cm^2^). The applied light pulses were controlled by an arbitrary function generator (Tektronix AFG 31000), and the illumination intensity was calibrated precisely using a silicon photodetector.

## 3. Results and Discussion

### 3.1. Synaptic Behavior by Optical Modulation in Biological/Artificial Visual System

**Scheme 1:**
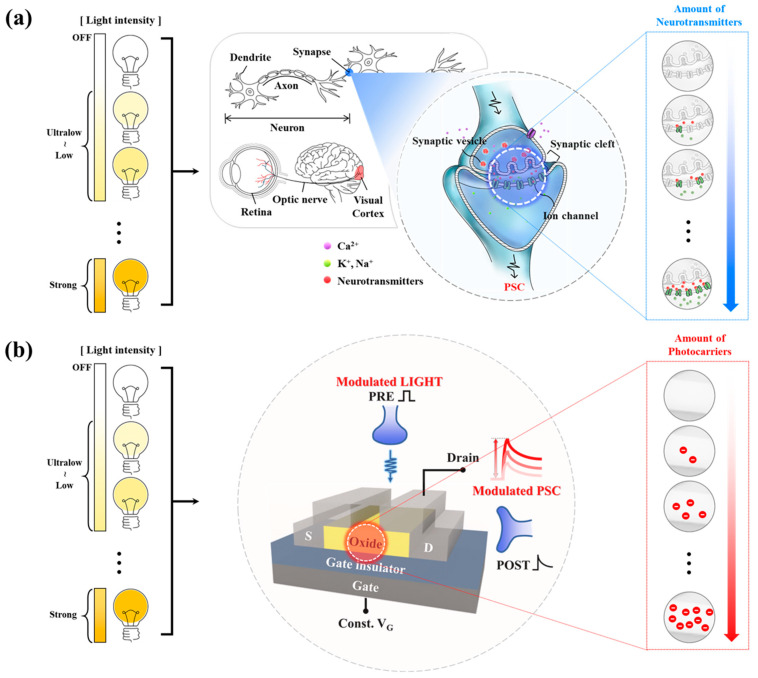
Mechanism of synaptic weight change for visual information processing: Schematic diagrams for (**a**) the control of neurotransmitters induced by optical signals in the biological synapse and (**b**) the artificial photonic synapse oxide TFT mimicking the biological synapse.

A general biological synapse receives the light of various wavelengths and intensities emitted or reflected from an object through the retina, which are converted into various types of electrical signals and transmitted to the brain, and then, visual perception executes the occurrence of information processing. At this time, for visual perception, such as the dispersion of color and complex patterns, the delicate processing of the optical signal transmitted to the brain is a very important step [29,30]. Scheme 1a describes the signal transduction system between the pre-neuron and post-neuron in detail during information processing of the biological synapse system. To transmit the received stimulus, the neurotransmitters (red), which have the most important role, transmit a signal to the synaptic cleft between neurons. When a nerve impulse reaches the presynaptic neuron and Ca^2+^ (purple) flows into the axon terminal simultaneously, neurotransmitters are released through rapid exocytosis and bind to the neurotransmitter receptor or ligand-gated ion channels, resulting in the transmission of electrical signals [3,25]. By controlling the number of emitted neurotransmitters, the electrical signal generated by the received stimulus opens ion channels by binding to a specific receptor; thus, diffusion results from the concentration gradient of ions such as K^+^ or Na^+^ (green) inside and outside the synapse and causes a change in synaptic weight. The number of neurotransmitters contains various pieces of information about the stimulus received from the outside, and information learning and memory processing are performed through signal transmission accordingly. Biological synapses can efficiently distinguish various wavelengths and intensities of stimuli by controlling the number of neurotransmitters formed by the received stimuli.

Currently, electronic devices with various structures are being used to imitate the continuous characteristics of the accepted stimulus of the biological synapse, and among them, a three-terminal transistor structure based on an amorphous oxide semiconductor (AOS) is being considered to function as a synaptic cleft. First, oxide semiconductors have high electron mobilities and very low off-current densities, enabling low power consumption and fast transfer rates similar to biological synapses [31,32]. In addition, owing to the existence of countless localized sub-gap defect states in the bandgap, a unique PPC phenomenon in which the changed conductance state is continuously maintained can be produced, even when the accepted stimulus is removed. Consequently, a long-term retention function can be implemented by simply inducing conductance change [14]. With the use of a three-terminal device, it is possible to maximize synaptic characteristics as well as lower power consumption by reducing off-current levels, which can be achieved by the formation of a depletion layer at the oxide/gate insulator interface under a continuous negative gate voltage. As described in Scheme 1b, for an ideal artificial synapse, the photocarriers generated by the intensity of the received stimulus mimic neurotransmitters of the biological synapse system. Thus, because the number of photocarriers due to a change in conductance determines the synaptic weight, previous studies applied materials with good absorbance to increase the photocarriers or various material combinations to increase photogeneration efficiency by reducing the recombination loss with the proper band alignment [14,33,34,35]. Although the artificial synapse devices studied have imitated the performance of biological synapses in various ways, a high-resolution system that can distinguish each successive synaptic weight is still extremely difficult, particularly when the received stimulus intensity is very low or ultralow.

### 3.2. Ideal Artificial Synapse Device for Optical Signal Detection

**Scheme 2:**
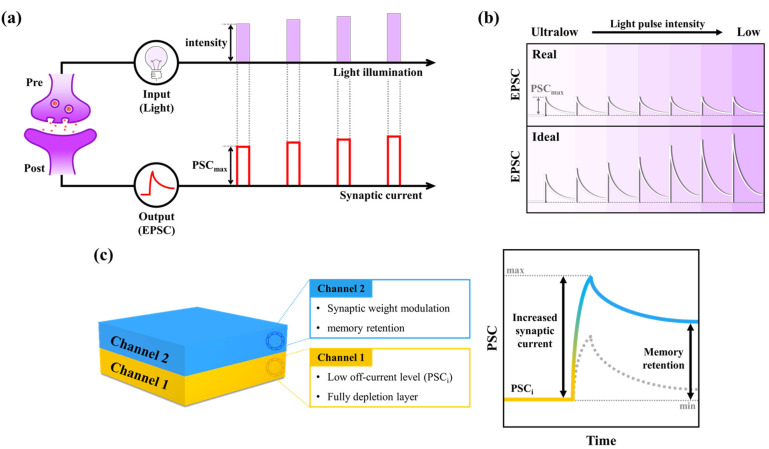
Schematic diagrams showing (**a**) ideal output (EPSC) characteristics under different input (light) intensities and (**b**) ideal/real synaptic current variations under modulating light intensities. (**c**) Effect of channel layer properties and stacking sequence on postsynaptic current variations.

Scheme 2a shows the change in the output EPSC according to the change in the input light when the photonic synapse can operate with a high-resolution system close to the biological synapse. Thus, the synaptic weight change expressed by the input light in the presynaptic neuron of the photonic synapse can be confirmed through the signal of the output EPSC, and the conductance change from different photocarrier generations causes a change in PSC_max_ at each output EPSC. Therefore, having different PSC_max_ values according to the stimulus intensity means that the operation of the photonic synapse can efficiently discriminate the synaptic weight change of the stimulus. The most important characteristic of an artificial synapse system is the variation of photocarriers produced by receiving external stimuli to generate a noticeable continuous change in the corresponding synaptic weight. However, because the photocarriers generated at ultralow intensities are extremely low, the change in synaptic weight is subtle, making it difficult to clearly distinguish the signals. Thus, although the received stimulus is continuous, the data extracted through information processing can be described continuously. Scheme 2b depicts real and ideal EPSC profiles when light pulses with intensities ranging from ultralow to low are incident on the photonic synapse. The ideal EPSC must represent a differentiated PSC_max_ even for a low-intensity stimulus, but the real EPSC does not demonstrate a change in the PSC_max_ signal with respect to ultralow light intensity. Thus, a dramatic change in synaptic weight must be implemented even at a continuous ultralow intensity with the following characteristics: (i) an extremely low current level (PSC_i_ state) before the external stimulus (fully depleted layer), (ii) an increase in the synaptic weight for successive stimuli, and (iii) continuous memory retention through the separation of the generated photocarriers. Previous studies presented results under high intensities of the received stimulus (>mW/cm^2^) and large intensity differences between the stimuli [14,17,18]. To increase the synaptic weight and lower the off-current level in the PSC_i_ state simultaneously, oxide semiconductor films with low conductivity must be preferentially used, and they must be sufficiently thin to form a full depletion layer with a low gate voltage. In contrast, oxide semiconductors with good conductivity are indispensable to maximizing synaptic weight and for the rapid transport of photocarriers generated from the stimuli.

Thus, we propose new oxide channels strategically utilizing each function by stacking two oxide semiconductors with different conductivities, resulting in the recognition of continuous ultralow-intensity signals from oxide synaptic transistors (Scheme 2c). Therefore, we fabricated a double-layer channel structure consisting of *a*-IGZO for a low PSC_i_ current and *a*-IZO with good conductivity for a high synaptic current and fast transport. In general, many reports on stacking structure channels have focused high electrical performances aiming at mobility enhancement. Our IGZO/IZO TFT used as a photonic synapse has the advantage of achieving appropriate channel conductivity with low off-current, synaptic weight change, and memory retention.

### 3.3. Electrical Characterization of a-IGZO and a-IZO TFTs

**Figure 1:**
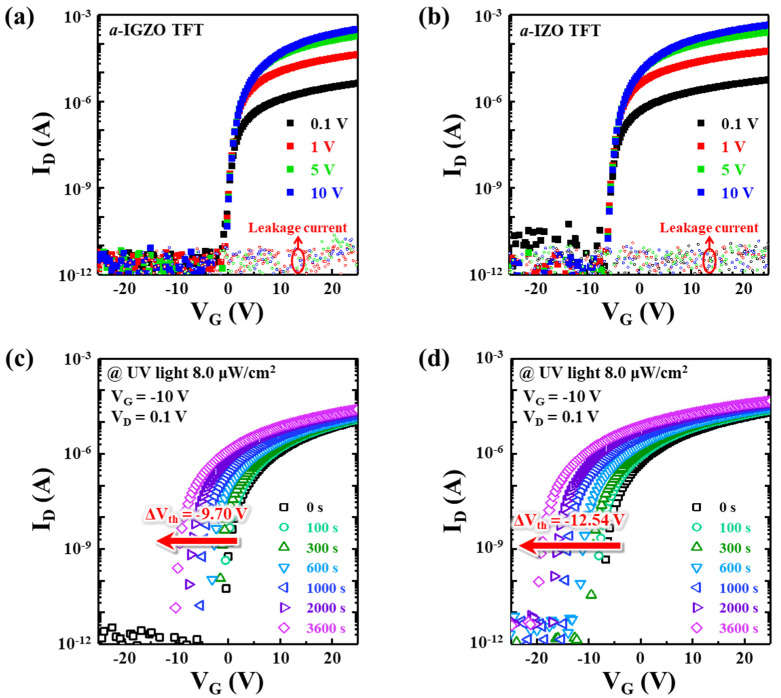
Electrical characteristics of the single-layer oxide TFTs: Transfer curves for (**a**) *a*-IGZO TFT and (**b**) *a*-IZO TFT. Negative V_th_ shift of transfer curves under the NBIS test with UV light (350 nm, 8.0 μW/cm^2^): (**c**) *a*-IGZO TFT and (**d**) *a*-IZO TFT.

Figure 1a,b show the transfer characteristics of the fabricated bottom-gate *a*-IGZO and *a*-IZO TFTs, measured at drain voltages (V_D_) of 0.1–10 V. The *a*-IGZO (In:Ga:Zn = 2:1:2) and *a*-IZO (In:Zn = 7:3) films were deposited with a thickness of 15 nm at Ar/O_2_ = 28:2 and post-thermally annealed at 200 °C for 1 h. As shown in Figure 1a, the *a*-IGZO TFT exhibited excellent transfer characteristics with a remarkably low off-current level of approximately 10^–12^ A, V_th_ of 0.18 V, SS of 0.22 V dec^−1^, μ_FE_ of 8 cm^2^ V^−1^ s^−1^, and on/off ratio of 10^8^. In addition, the *a*-IGZO TFT with a considerably low SS value can be switched from the off-state to the on-state with only a low V_G_. We extract the interface defect density (*D_it_*) using Equation (1):

(1) $$D_{it}=\frac{C_{i}}{q^{2}}\left(\frac{q\cdot SS}{kTln10}-1\right),$$

where *C_i_* is the capacitance of the dielectric, *q* is the magnitude of electron charge, *k* is the Boltzmann constant, and *T* is temperature in Kelvin. As mentioned above, the SS value of the *a*-IGZO TFT is 0.22 V dec^−1^, and the calculated *D_it_* is 2.9 × 10^11^ cm^−2^ eV^−1^. The small *D_it_* value indicated excellent interfacial properties. Furthermore, the V_th_ shift in hysteresis characteristic demonstrating the interface states was negligible (Figure S1a). As shown in Figure S1b, the positive V_th_ shift of the *a*-IGZO TFT under positive bias stress for 3600 s was as small as 3.0 V. Different types of metal cations have different oxygen affinities, which cause different chemical bonds between the metal and oxygen, affecting the carrier concentration in the channel layer. The gallium (Ga) ions in the *a*-IGZO compound have a higher oxygen affinity than indium (In), which provides strong chemical metal–oxygen bonding, thereby reducing the amount of excess free carriers generated through V_O_ compared with *a*-IZO with high In content [36]. In contrast, the performance values of the *a*-IZO TFT with high carrier concentration were V_th_ = −5.39 V, SS = 0.27 V dec^−1^, μ_FE_ = 16.7 cm^2^ V^−1^ s^−1^, and on/off ratio = 10^7^. Compared with the *a*-IGZO TFT, it had a relatively high mobility but exhibited high off-current level characteristics of approximately 10^–11^ A (Figure 1b). Although the *a*-IGZO and *a*-IZO films were prepared at the same low temperature of 200 °C, they exhibited noticeable differences in TFT performances owing to their inherently different conductivities. In particular, the high carrier density of the *a*-IZO TFT resulted in a decrease in the on/off ratio (the difference of one order of magnitude in the off-current level) and a large negative shift of V_th_. In contrast, typical oxide-based TFTs exhibit considerable changes in transfer characteristics owing to continuous stress conditions, such as bias and illumination [37]. In particular, the photosensitivity of visible light has been considered a major technical challenge to be resolved for production and differs depending on the type of AOS used for the channel layer. Thus, we performed negative-bias illumination stress (NBIS) stability tests for single *a*-IGZO and *a*-IZO TFTs under a negative V_G_ (−10 V) and ultraviolet (UV) light illumination (8.0 μW/cm^2^). Figure 1c shows the variation in the transfer curves of the *a*-IGZO TFT when UV light was illuminated for 3600 s under a constant V_G_ of −10 V at room temperature. As the light stress time increased, V_th_ shifted continuously to the negative side, and the V_th_ shift (∆V_th_) of approximately −9.7 V occurred for 3600 s. Similar to the *a*-IGZO (≈3.5 eV), most AOSs have a wide bandgap, but such NBIS instability is due to the existence of high-density subgap states located close to the valence-band minimum (VBM) of AOSs [37]. The existence of defects such as V_O_ and weakly bonded oxygen induces the photo-instability of AOS even with long-wavelength visible light of low photon energy by providing an effective optical gap (EOG) that is narrower than the actual optical bandgap [38]. The NBIS stability test of *a*-IZO TFT is also shown in Figure 1d, and ∆V_th_ using UV light for 3600 s was −12.5 V, which indicated a remarkably higher shift than *a*-IGZO TFT under the same stress condition, indicating lowered photo-stability. Since *a*-IZO has a relatively narrower bandgap and larger defect density, we can expect that photoexcitation by UV illumination proceeds more actively with a large ∆V_th_ value in the NBIS stability test. These ∆V_th_ behaviors for *a*-IGZO and *a*-IZO TFTs can be effectively used to obtain dramatic synaptic current changes to the photonic synapse as a presynaptic stimulus under light illumination.

### 3.4. EPSC Characteristics of a-IGZO and a-IZO TFTs

**Figure 2:**
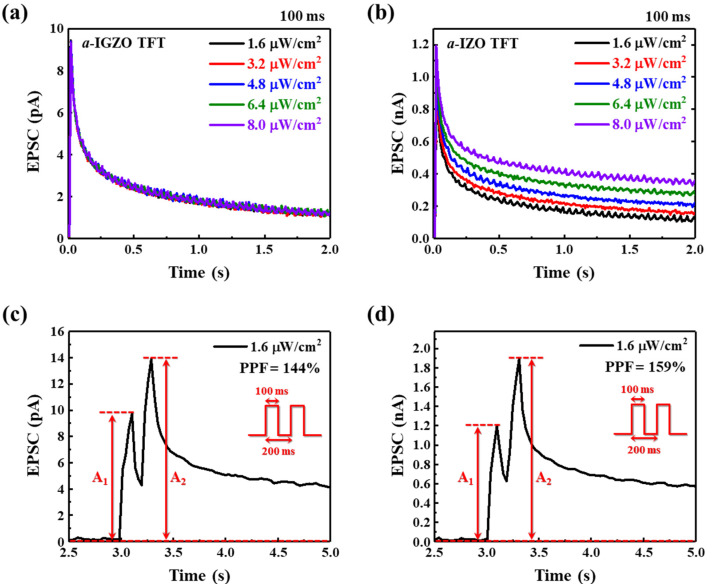
Photonic performances of the TFTs irradiated with UV light (1 pulse) of varying light intensities: (**a**) *a*-IGZO TFT and (**b**) *a*-IZO TFT. The EPSC data for (**c**) *a*-IGZO TFT and (**d**) *a*-IZO TFT stimulated with identical UV light pulses with time intervals of 200 ms.

Consequently, the negative shift in ∆V_th_ causes an abrupt change in the transfer characteristic from the off-state region to the subthreshold region under a constant V_G_, and simultaneous light illumination can result in an exponential increase in the synaptic current. Therefore, to clearly distinguish light stimuli within the ultralow intensity range, we strategically induced a significant change in the transfer characteristics from the off-state to the subthreshold region, promoting an effective increase in the synaptic current. As shown in Figure 2a,b, we designed an applied voltage of V_G_ = −10 V and external pulse stimulation to 350 nm UV light, and the EPSC characteristics of the photonic synapse transistors with *a*-IGZO and *a*-IZO single channels were evaluated in the ultralow intensity range. Here, a light of 1.6–8.0 μW/cm^2^ was irradiated with very fine intensity intervals of 100 ms. As shown in Figure 2a, when ultralow intensity light was applied, the photocurrent of the *a*-IGZO synaptic transistor exhibited almost the same at ≈10 pA level despite the difference in light intensity. This indicated that subtle changes in the light intensity were not distinguished by the synaptic weight. Thus, we confirmed that the *a*-IGZO TFT has the advantage of a very low PSC_i_ owing to a considerably low off-current level but cannot achieve a noticeable increase in the synaptic current under ultralow-intensity light. In addition, the memory retention of the PPC phenomenon was almost identical. In contrast, for the *a*-IZO TFT (Figure 2b), we observed that the change in synaptic weight was relatively clear, and memory retention on light intensity was distinct, which was attributed to the high conductivity of the *a*-IZO and large V_th_ shift through light illumination. However, this high conductivity resulted in a high off-current level (high PSC_i_) before light illumination. The high off-current level of the *a*-IZO TFT slightly attenuated the effect of increasing the synaptic current through the V_th_ shift by the applied bias and light stress.

Thus, the capability of detecting continuous signals under exposure to ultralow intensity light with fine intervals is closely related to the conductivity of the channel layers. One of the most important synaptic functions in the memory behavior of biological synapses is PPF, which is a typical memory characteristic in which the PSC of the next stimulus is effectively increased by the previous stimulus when the next stimulus is input close to the previous stimulus. This PPF can be defined as the ratio of the amplitude between the first and second PSCs, and it can be described by Equation (2) [25]:

(2) $$PPF=\left(\frac{A_{2}}{A_{1}}\right)\times 100\%,$$

where *A*_1_ and *A*_2_ denote EPSCs expressed by the first and second applied light pulses, respectively, and they are calculated through the difference between PSC_i_ and PSC_max_ increased by each light pulse. The PPF mimicked by the *a*-IGZO TFT under UV light pulses of ultralow intensity (1.6 μW/cm^2^) is shown in Figure 2c. The same two UV light pulses were incident on the *a*-IGZO channel at a time interval (Δt) of 200 ms, and the synaptic current was read with a V_D_ of 0.1 mV. The EPSC increased when the second UV light pulse was higher than the first one, and the calculated PPF of the *a*-IGZO TFT was 144%. The EPSC changes induced by continuous pulses with a short interval time were very similar to the characteristics of short-term synaptic behaviors observed in biological synapses. The PPF value of the *a*-IZO TFT under the same measurement conditions was 159%, which was relatively higher than that of the *a*-IGZO TFT (Figure 2d). The improvement in the PPF characteristics can be attributed to the increase in the second EPSC peak, which was influenced by the degree of current retention of the first EPSC peak.

### 3.5. Modulation of the Ar/O_2_ Ratio for Enhancing Photonic Performances

**Figure 3:**
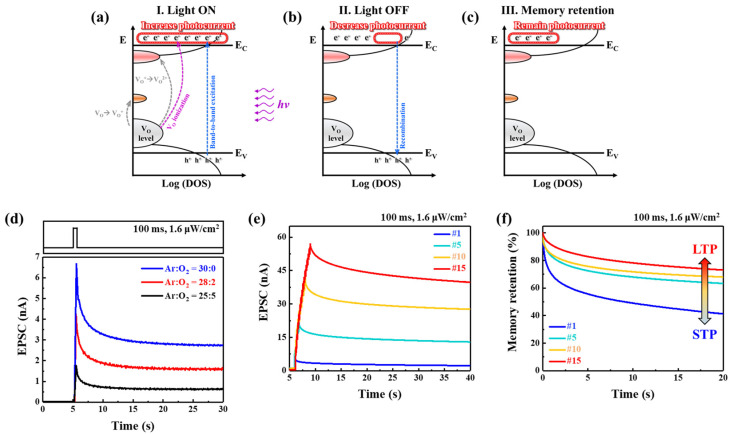
Schematic band diagrams of oxide semiconductors under (**a**) light ON, (**b**) light OFF, and (**c**) retention conditions. (**d**) Variation in EPSC signals from the TFTs with the *a*-IZO films prepared using different Ar/O_2_ ratios. (**e**) EPSC characteristics by pulse number variation from the IGZO/IZO (Ar/O_2_ = 30:0 for *a*-IZO) synaptic transistors with a light pulse width of 100 ms. (**f**) STP and LTP behaviors of memory retention caused by different pulse numbers.

From these EPSC characteristics of the *a*-IGZO and *a*-IZO TFTs under ultralow intensity illumination, an alternative channel layer structure that enables both significant synaptic weight change and memory behavior should be suggested to perform an effective synaptic function as a synaptic transistor. In particular, the change in the synaptic weight of the photonic synapse transistor is predominantly determined by the photocarrier generation and recombination process of the channel layer in the on/off states of the applied light. The photocarrier dynamics in the channel layer are divided into light illumination (light ON), light removal (light OFF), and time elapsed (memory retention) stages. First, in the light ON stage, photocarriers are created by electron–hole pair generation and ionization of oxygen defects, inducing an abrupt change in the synaptic weight via a rapid increase in conductance (Figure 3a). Thus, to induce a large change in the synaptic weight, the conductance should be increased and read quickly via more photoexcitation reactions. As shown in Figure 3b, after the light was removed, the generated photocarriers disappeared through the recombination process, and the conductance of the channel gradually decreased. However, the photocarriers related to the ionized V_O_ contributing to the PPC phenomenon were still located in the conduction band even after a certain period had elapsed; thus, it exhibited a retention phenomenon that did not return to the PSC_i_ current, which is defined as memory retention, indicating memory-like behavior (Figure 3c). This memory modulation is an important factor in the neuromorphic system, and as the number of defects in the oxide semiconductor increases, it can result in PPC characteristics that are advantageous for realizing non-volatile memory. Finally, we observed that the non-volatile synaptic plasticity function can be successfully implemented when the increased EPSC was retained via V_O_ photoionization based on a low PSC_i_ and the considerable generation of photocarriers. To fully satisfy these requirements, bilayer stacking synaptic transistors can demonstrate both synaptic weight change and memory behavior with excellent characteristics by effectively using the advantageous functions between *a*-IGZO and *a*-IZO films. As shown in Figure S2a–c, the transfer characteristics of the IGZO/IZO TFT exhibited good performances and the negative shift of V_th_ as the Ar/O_2_ ratio increased. Compared with the results of *a*-IGZO TFT (Figure 1a), the proposed IGZO/IZO TFT exhibited the enhanced mobility as well as the maintenance of low off-state current. The IGZO/IZO TFT fabricated at Ar/O_2_ of 30/0 showed the best performance with SS of 0.27 V dec^−1^ and μ_FE_ of 12.4 cm^2^ V^−1^ s^−1^ (Figure S2d). Although the *a*-IZO with relatively high conductivity was used in the stacked structure, the electrical mobility exhibits an appropriate value between the performances of *a*-IGZO and *a*-IZO TFTs. In addition, the TFT devices often suffer from anomalous hump phenomenon in subthreshold region of transfer curves. This behavior is closely related to the charge traps at the interface, resulting in the degradation of SS. However, the IGZO/IZO TFT showed a steep SS without deterioration despite the bilayer stacking of two channel materials. Consequently, it was found that the *a*-IZO contributed to the improvement of the conductivity in the stacked channel, and the *a*-IGZO served as the main channel for charge transport. Figure 3d shows the variation in the EPSC signals according to the Ar/O_2_ ratio during *a*-IZO deposition of the IGZO/IZO double layer. Here, the Ar/O_2_ ratio was set to 30/0, 28/2, and 25/5, and all three PSC characteristics revealed a very low off-current level. This result indicated that the *a*-IGZO channel layers were fully depleted under the gate voltage conditions. Since a decrease in the oxygen partial pressure increases the V_O_ concentration of the *a*-IZO film, it can induce more photoionization and stronger PPC under the same light stimuli. In addition, the EPSC characteristics of the IGZO/IZO TFT exhibited a higher synaptic current under light illumination and a higher remaining current in the light OFF stage as the oxygen partial pressure in the *a*-IZO deposition decreased. As a result, for the IGZO/IZO bilayer TFT, the bottom layer is used to attain the low off-current, and the top layer plays a key role for effective charge transport. A large amount of V_O_ in the channel was effectively ionized by UV light, and numerous photocarriers were released, leaving the remaining photocarriers even after the light OFF owing to the strong PPC phenomenon, causing an increase in memory characteristics. We obtained a very low PSC_i_ current through the low-conductivity *a*-IGZO and achieved an effective improvement in synaptic current and memory characteristics by controlling the Ar/O_2_ ratio of the high-conductivity *a*-IZO. This memory behavior can be enhanced by intensifying synaptic connections via repeated stimulation, which is strongly affected by the number and frequency of external stimulations. Figure 3e shows the EPSC changes depending on the pulse number for the IGZO/IZO TFT (Ar/O_2_ = 30/0 for *a*-IZO), indicating an effective synaptic current increase. The pulsed UV illumination conditions (350 nm, 1.6 μW/cm^2^, w: 100 ms, Δt: 200 ms) were identical, and the pulse numbers were measured from 1 to 15. When pulse number 1 was applied to the synaptic transistor, the synaptic current temporarily increased and then rapidly decreased. However, as the stimulus was repeatedly applied at 200 ms intervals, photogenerated carriers in the channel could not be fully recombined because of the contribution of the strong PPC effect, and the synaptic current continuously increased with repeated accumulation. In addition, although the current gradually decreased after light OFF, sufficient memory retention with a high synaptic current was clearly detected, even after a long period. The characteristics of consistently maintaining synaptic weight produced by repetitive stimuli are ultimately similar to LTP, in which stimulus information is stored for a long time [39]. Consequently, successive pulses applied at a high rate induce non-volatile memory characteristics. The decrease in the information forgetting rate and memory enhancement depending on the repetitions of stimulation means that the IGZO/IZO double-layer TFT can be applied as a synaptic device that can mimic human brain memory. The memory characteristics of a synaptic device influenced by the pulse number can be quantitatively analyzed using memory retention (*M_t_*). *M_t_* is normalized to the synaptic current after the stimulus is removed. By comparing the amount of remaining synaptic current for several EPSCs measured under different conditions, the non-volatile memory characteristics can be identified using Equation (3) [39]:

(3) $$M_{t}=\left(PSC_{t}-PSC_{i}\right)/\left(PSC_{max}-PSC_{i}\right),$$

where *PSC_t_* is the postsynaptic current at a specific time t, *PSC_i_* is the initial value of the current without light stimulation, and *PSC_max_* is the highest value of the postsynaptic current. Figure 3f shows a continuous increase in *M_t_* with an increase in the pulse number. When passing 20 s after the decay of synaptic current, a one-time pulse input of a 100 ms UV light (350 nm, 1.6 μW/cm^2^) exhibited a relatively low *M_t_* of 41%. Pulse number 1 exhibited a rapid current decrease compared with the decay characteristics of the EPSC from the samples experiencing multiple pulse numbers of >1. However, when the 15th pulse number was applied, it exhibited a high *M_t_* of approximately 73% under the same 20 s condition. Thus, the volatile and non-volatile characteristics of memory can be distinguished using pulse numbers. When an external light stimulus is provided at short intervals, the PPC effect in photonic synapses depends on the repetition number of the stimulus and affects memory behavior.

### 3.6. Emulation of Synaptic Behavior with IGZO/IZO Photonic Synapse Transistor

**Figure 4:**
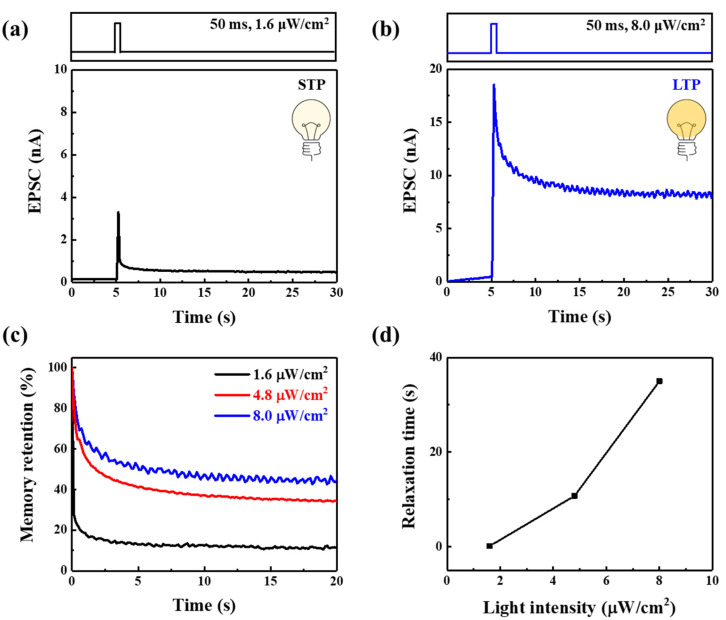
EPSC characteristics of the IGZO/IZO (Ar/O_2_ = 30:0 for *a*-IZO) TFT under different modulating intensities: (**a**) The short-term plasticity (STP) and (**b**) long-term plasticity (LTP) behaviors caused from UV light of 1.6 and 8.0 μW/cm^2^. (**c**) Memory retention with EPSC decay characteristics responding to a series of increasing light intensity. (**d**) Comparison of the relaxation time as a function of light intensity.

As shown in Figure 4a–d, we attempted to confirm the change in synaptic weight in the IGZO/IZO synaptic transistors with respect to the light intensity, where ultralow intensity light was used as an external stimulus. Figure 4a shows the result of applying a single light pulse of ultralow intensity (1.6 μW/cm^2^) for a very short time of 50 ms to the device with a double-layer structure, obtaining $\;PSC\left(=PSC_{max}-PSC_{i}\right)$ of 3.1 nA. For a single light pulse of low intensity of 8.0 μW/cm^2^, a high synaptic current corresponding to ∆PSC of 18.1 nA was induced (Figure 4b). Despite maintaining an extremely low PSC_i_ with the input of ultralow-intensity light, a high synaptic current increase resulted in a change in the synaptic weight. As a result, the suggested IGZO/IZO TFT with double-layer structure exhibited synaptic performances of excellently reading input light signals with an ultralow intensity range, and memory characteristics with distinct signal differences were also achieved. The dependence of *M_t_* on light intensity is shown in Figure 4c. The memory level of the synaptic transistor was effectively driven by controlling the light intensity. The result under the lowest light input of 1.6 μW/cm^2^ illustrated that the increased current level returned very close to the initial value of PSC_i_, indicating STP characteristics similar to the “volatile-like” behavior of the human brain. However, as the light intensity increased, LTP characteristics indicating a slow current decrease were observed. STP and LTP behaviors are considered as the key factors in the memory performance of synapse devices. The change in synaptic current from rapid to slow decay according to the light intensity indicates that the IGZO/IZO TFT can mimic the transition from STP to LTP. Meanwhile, memory retention representing EPSC decay follows the Kohlrausch stretched exponential function, as shown in Equation (4) [40]:

(4) $$I_{D}\left(t\right)=I_{D}\left(0\right)\times \exp\left[-{\left(t/\tau \right)}^{\beta }\right],$$

where *I_D_* (*t*) and *I_D_* (0) are the current levels at times t and t = 0, respectively, β is the stretch index in the range between 0 and 1, and τ is the relaxation time constant that can be used to determine the forgetting rate. As shown in Figure 4d, each relaxation time is presented together when the light of ultralow intensity from 1.6 to 8.0 μW/cm^2^ is provided as a single pulse. For 1.6 μW/cm^2^ exhibiting a relatively low *M_t_*, a short τ value of 0.12 s was calculated, resulting in a rapid decrease rate close to PSC_i_. In contrast, when the light with 8.0 μW/cm^2^ was irradiated, the synaptic current decreased at a slow speed and revealed a long relaxation time of τ = 35 s.

## 4. Conclusions

We have strategically proposed bilayer IGZO/IZO photonic synapse transistors with a functional stacking structure that can precisely control the synaptic weights of various optical signals. Designing a combination of oxide semiconductor layers with different electrical behaviors enables dramatic synaptic weight changes to be achieved by allocating the unique role of each channel. Even under an ultralow-intensity light of 1.6 μW/cm^2^, the synaptic transistors distinctly exhibited a high-resolution EPSC signal with low PSC_i_ and excellent memory retention by effectively using advantageous functions of *a*-IGZO and *a*-IZO films. In addition, the fundamental synaptic function was successfully emulated in response to various optical stimuli such as EPSC, PPF, STP, and LTP. Furthermore, the decrease in the information forgetting rate and memory enhancement depending on the repetitions of stimulation implied that the IGZO/IZO transistors revealed their potential as a synaptic device capable of mimicking the function of the human brain memory. The proposed IGZO/IZO photonic synapse transistors are expected to create the foundation for the development of next-generation neuromorphic computing systems for visual information processing.

## Supplementary Materials

The following supporting information can be downloaded at: https://www.mdpi.com/article/10.3390/mi13040526/s1, Figure S1: (a) Hysteresis characteristics of *a*-IGZO TFT measured under forward (−30 V to 30 V) and reverse (30 V to −30 V) sweeping. (b) Transfer characteristics of *a*-IGZO TFT under continuous positive bias stress (PBS) with V_G_ of 30 V; Figure S2: Transfer characteristics of the IGZO/IZO TFTs under various Ar/O_2_ ratio: (a) Ar/O_2_ = 25/5, (b) Ar/O_2_ = 28/2, (c) Ar/O_2_ = 30/0. (d) Summary of electrical performances of the IGZO/IZO TFT.
