# Supplementary material for: Design of Functionally Stacked Channels of Oxide Thin-Film Transistors to Mimic Precise Ultralow-Light-Irradiated Synaptic Weight Modulation
Source: Micromachines (Basel). 2022 Mar 26;13(4):526. doi: 10.3390/mi13040526 (PMC9031837; doi:10.3390/mi13040526)
Supplement: Supplementary file 1 [file micromachines-13-00526-s001.zip › micromachines-1659909-supplementary.pdf]

micromachines

MDPI

Article

Design of Functionally Stacked Channels of Oxide Thin-Film Transistors to Mimic Precise Ultralow-Light-Irradiated Synaptic Weight Modulation

Ji Sook Yang [1,†], Sung Hyeon Jung [1,†], Dong Su Kim [1], Ji Hoon Choi [1], Hee Won Suh [1], Hak Hyeon Lee [1], Kun Woong Lee [1] and Hyung Koun Cho [1,2,*]

[1] School of Advanced Materials Science and Engineering, Sungkyunkwan University (SKKU), 2066 Seobu-ro, Jangan-gu, Suwon 16419, Gyeonggi-do, Korea; yangtwin1@naver.com (J.S.Y.); wjdtjdgus2@skku.edu (S.H.J.); dskim2846@naver.com (D.S.K.); jeehun_choi@naver.com (J.H.C.); naekkeo@skku.edu (H.W.S.); zadxs@skku.edu (H.H.L.); leekunwoong1@naver.com (K.W.L.)

[2] Research Center for Advanced Materials Technology, Sungkyunkwan University (SKKU), 2066 Seobu-ro, Jangan-gu, Suwon 16419, Gyeonggi-do, Korea

* Correspondence: chohk@skku.edu; Tel.: +82-31-290-7364

† These authors contributed equally to this work.

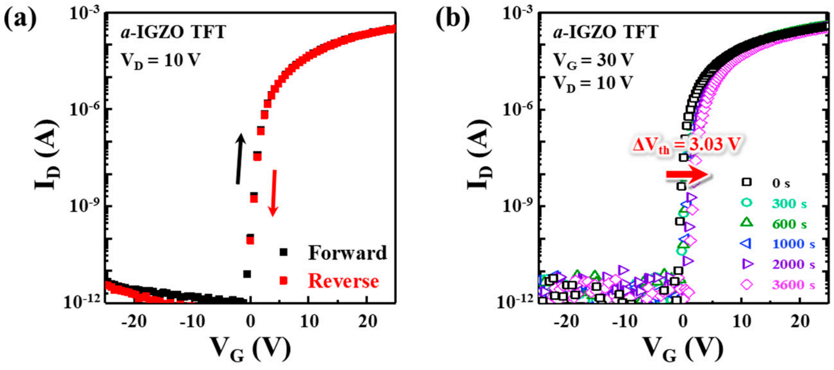

Figure S1. (a) Hysteresis characteristics of *a*-IGZO TFT measured under forward (-30 V to 30 V) and reverse (30 V to -30 V) sweeping. (b) Transfer characteristics of *a*-IGZO TFT under continuous positive bias stress (PBS) with V_G of 30 V.

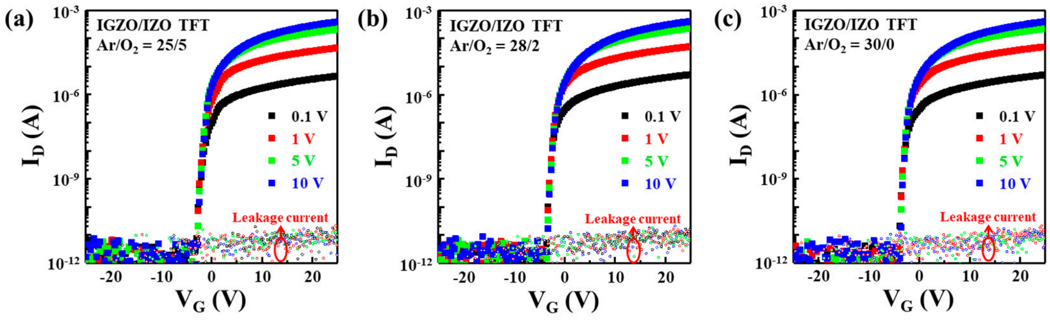

(d)

	Ar/O_2 = 25/5	Ar/O_2 = 28/2	Ar/O_2 = 30/0
V_{th}	-3.4 V	-3.8 V	- 4.1 V
SS	0.35 V dec^{-1}	0.29 V dec^{-1}	0.27 V dec^{-1}
μ_{FE}	10.1 cm^2 V^{-1} s^{-1}	11 cm^2 V^{-1} s^{-1}	12.4 cm^2 V^{-1} s^{-1}

Figure S2. Transfer characteristics of the IGZO/IZO TFTs under various Ar/O_2 ratio: (a) Ar/O_2 = 25/5, (b) Ar/O_2 = 28/2, (c) Ar/O_2 = 30/0. (d) Summary of electrical performances of the IGZO/IZO TFT.
